# Glances at the Hospitals

**Published:** 1900-10-20

**Authors:** 


### GLANCES AT THE HOSPITALS.

### LIVERPOOL ROYAL INFIRMARY.

The ordinary receipts amounted to £11,297 for the year 1899, and the ordinary expenditure to £14,060. There is a total debt against the institution of £3,391. We failed to find any statement relative to the cost per patient or to the cost per bed occupied. The report states that, for the first time in the history of the hospital, a claim for poor and city rates has been made and enforced. And it is added that an institution carrying on a work which would otherwise fall on the ratepayers should be compelled to pay £162 per annum from money procured with difficulty seems indeed a hardship. Probably every one of our readers will agree that it is a hardship, and will hope that the Liverpool committee will obtain remission of this unfair charge. The hospital contained on January 1st, 1899, 258 patients: 3,012 were admitted during the year, making a total of 3,270. Of these 2,706 were discharged; 310 died; and 254 remained in the house on the last day of December. The death rate was 10·28 per cent.; but excluding 38 who died within 24 hours of admission it would be reduced to 9·14 per cent. The medical and surgical tables are drawn out with much care. Every disease named has placed opposite it the numbers, male and female, suffering from it; the ages of the patients; and the numbers cured, relieved, not relieved, transferred, and died. The table of operations gives similar information. In this table it is stated that the operation for appendicitis was performed on 18 men and 7 women, and that 12 were cured and 5 died, which numbers make 17 only; 57 amputations were performed. In 50 of these cases cure followed; in 5 relief; and in 2 death. It would be desirable to add an additional column showing the immediate cause of death, and giving "remarks," as in the gynæcological table. Ovariotomy was performed 13 times, and in all cases a cure followed. In the ophthalmic department 91 operations were performed; but here, oddly enough, we do not get the results. The Röntgen rays were used 180 times. There is no table showing the anæsthetic used in the operations. As the experience of the Liverpool Infirmary surgeons is necessarily a very wide one, such information would have been most valuable.

### THE HULL ROYAL INFIRMARY.

The total ordinary receipts of this hospital for the year 1899 amounted to £8,343, and the expenditure to £8,804. The year opened with a balance at the bank of £1,025, but the debts amounted to £880. The year closed with the balance of £85, but the debts on the general account amounted to £909, and on the building account to £174. The committee state that the largely-increased demand made by in-patients on the resources of the hospital, coupled by the want of increased subscriptions, is causing them much anxiety; and on referring to the table on page 22 we find that the annual subscriptions amounted to only £2,125, but the donations from operatives amounted to £1,974. That is, almost as much as the annual subscriptions. It is hardly creditable to the inhabitants of a large and wealthy place like Hull that the annual subscriptions should fall so low. The average number of beds occupied was 160, and the average duration of each patient's stay in the hospital was about 22½ days. The average cost of each in-patient for treatment, nursing, and maintenance was £2 10s.; the cost per week per patient was 15s. 7¾d.; and the average cost per bed occupied was £47 12s. In the out-patients' department there were 78,475 attendants, and the cost was a little over 1s. per patient. The number of in-patients was 1,597. This is one of the very few hospital reports that makes its appearance without medical or surgical statistics.

MANCHESTER CLINICAL HOSPITAL.

The Committee say that the ordinary income of the infirmary shows a tendency to decrease year by year, while the expenditure, with every regard to economy, seems to increase. This is certainly a most unsatisfactory state of affairs; and unless the income can be increased or the expenditure lessened the Committee will have to part with some of their invested funds, a course which they are naturally reluctant to take. The annual subscriptions only amounted to £929, and it does not appear from the statement given on page 12 of the Report what portion of this was contributed by the working people themselves. The deficit on the year's working was £1,129, and £890 has to be added to that as remaining from the previous year. The Report of the Committee is not so full as it might be. We do not see any statement of the cost per head per week nor the cost per bed occupied. The in-patients reached a total of 1,122, being an increase of 70 as compared with the number for 1898. A total of 630 operations were performed, 457 of these being on children, and in addition to these there were 114 performed in the out-patients' department, in which department there was a total of 9,593 new cases. The tables are satisfactory. It is easy to follow the cases to their terminations in recovery, relief, or death. For instance, there were 78 cases of pneumonia, and of these 58 recovered, 16 died, and 4 were under treatment on the date of the Report. The hospital is intended for women and children only, and, as far as women patients are concerned, chiefly gynæcological cases are received.

THE TAUNTON AND SOMERSET HOSPITAL.

The general income for 1899 amounted to £3,356, and the general expenditure to £5,180. This large deficiency is causing the committee "extreme anxiety," and they attribute the fact partly to the exceptional expenditure which has been incurred in repairs and to the reduction in the amount received as donations. No doubt these will account for much of the excess of expenditure over income, but it must always be a matter for regret that any infirmary committee should sanction any extraordinary expenditure unless they actually possess the money to meet such expenditure. It is with institutions as with individuals; the balance of sixpence should always be on the right side. When an infirmary committee finds itself in this position it has three courses open to it:—First, it can make strenuous efforts to increase its annual subscriptions; second, it can reduce expenditure by economical management, if such reduction can be made without sacrificing efficiency; and third, it can reduce the number of beds occupied. There were 85 patients in residence on January 1st, 1899, and 74 on December 31st. During the year 798 patients were admitted. Of these, 561 were discharged cured or relieved, 29 not relieved, 66 for other reasons, and 47 died. The daily average number resident was 81; and the average duration of residence was 35 days. The out-patients numbered 3,988. The information given in the medical report is extremely meagre, and no conclusions can be drawn from it, beyond the bald figures already given.

